# Self-induced superradiant masing

**DOI:** 10.1038/s41567-025-03123-0

**Published:** 2026-01-02

**Authors:** Wenzel Kersten, Nikolaus de Zordo, Oliver Diekmann, Elena S. Redchenko, Andrew N. Kanagin, Andreas Angerer, William J. Munro, Kae Nemoto, Igor E. Mazets, Stefan Rotter, Thomas Pohl, Jörg Schmiedmayer

**Affiliations:** 1 Vienna Center for Quantum Science and Technology, Atominstitut, Vienna University of Technology (TU Wien), Vienna, Austria; 2 Institute for Theoretical Physics, Vienna University of Technology (TU Wien), Vienna, Austria; 3 Okinawa Institute of Science and Technology Graduate University (OIST), Okinawa, Japan

**Keywords:** Quantum optics, Condensed-matter physics, Lasers, LEDs and light sources

## Abstract

In cavity quantum electrodynamics and particularly superradiance, emitters are typically assumed to be independent, interacting only through light shared via a common mode. Although such photon-mediated interactions lead to a wide range of collective optical effects, direct dipole–dipole interactions within the emitter ensemble are generally viewed as a source of decoherence. Here we report the role of direct spin–spin interactions as a drive for the superradiant dynamics of a hybrid system of nitrogen-vacancy centre spins in a diamond coupled to a superconducting microwave cavity. After an initial fast superradiant burst, we observe a train of subsequent emission pulses followed by quasi-continuous masing for up to one millisecond. We show that this behaviour arises from spectral hole refilling, where spin inversion is redistributed into the superradiant window of spins resonant with the cavity. We report measurements that exclude other cavity-related effects and perform microscopic simulations that confirm that the observed behaviour is driven by dipole–dipole interactions between the spins. These findings open pathways for exploring complex spin–spin interactions in dense disordered systems and offer possibilities for ultranarrow-linewidth solid-state superradiant masers powered purely by microwave-driven spin control.

## Abstract (web summary)

Superradiance is usually driven by light-mediated couplings, leaving the role of direct emitter interactions unclear. Now, it is shown that dipole–dipole interactions in diamond spins drive self-induced pulsed and continuous superradiant masing.

## Main

Superradiance, first predicted by Dicke in 1954 (ref. ^1^), describes an enhanced collective emission of light exhibiting high coherence and a nonlinear scaling of intensity with the number of emitters^2,3^. It has since been observed in many systems^4–8^ including solid-state realizations with quantum dots^9,10^ and the negatively charged nitrogen-vacancy (NV^−^) centres in diamond^11,12^. This collective effect lies at the heart of cavity quantum electrodynamics^13^, where emitters are typically treated as non-interacting and their mutual couplings are mediated solely through the shared cavity field. These light-mediated interactions give rise to a variety of collective phenomena and phase transitions^14–17^.

Building on these fundamental explorations of collective light–matter interactions, recent advances have shifted the focus towards their applications in quantum technologies^18,19^. Among other solid-state platforms^20–22^, diamond-based systems stand out due to the exceptional quantum coherence and optical controllability of NV^−^ centres^23^. Key examples include diamond-based microwave amplifiers^24^, quantum sensors^25^, mode cooling platforms^26,27^ and room-temperature diamond masers^28^. Operating a diamond maser in a superradiant regime could further narrow its linewidth, with coherence maintained by the high cooperativity of the cavity–spin system itself^29^, rather than being dictated by the intracavity photon count, as that in conventional masers^30^. This could pave the way for ultranarrow-linewidth sources, enabling high-precision frequency generation and quantum-limited microwave amplification. However, achieving a continuous-wave superradiant diamond maser remains an open challenge due to the need for both strong and uniform collective spin–photon coupling along with efficient optical pumping^31^. Although increasing the density of the spin ensemble enhances coupling, it also reduces optical transparency, complicating efficient optical pumping; moreover, it introduces greater dipole–dipole interactions, which can lead to decoherence^32,33^. In this work, we show that such interactions can take on a more constructive role in dense spin systems like NV^−^ centres. Our measurements reveal that dipole–dipole interactions can actively drive superradiant masing, which opens up a new self-induced regime of coherent collective emission in solid-state systems. We perform a comprehensive experimental characterization of the effect and conclusively trace it back to dipole–dipole interactions by numerically simulating the microscopic dynamics of up to one million NV^−^ centres.

**Fig. 1:**
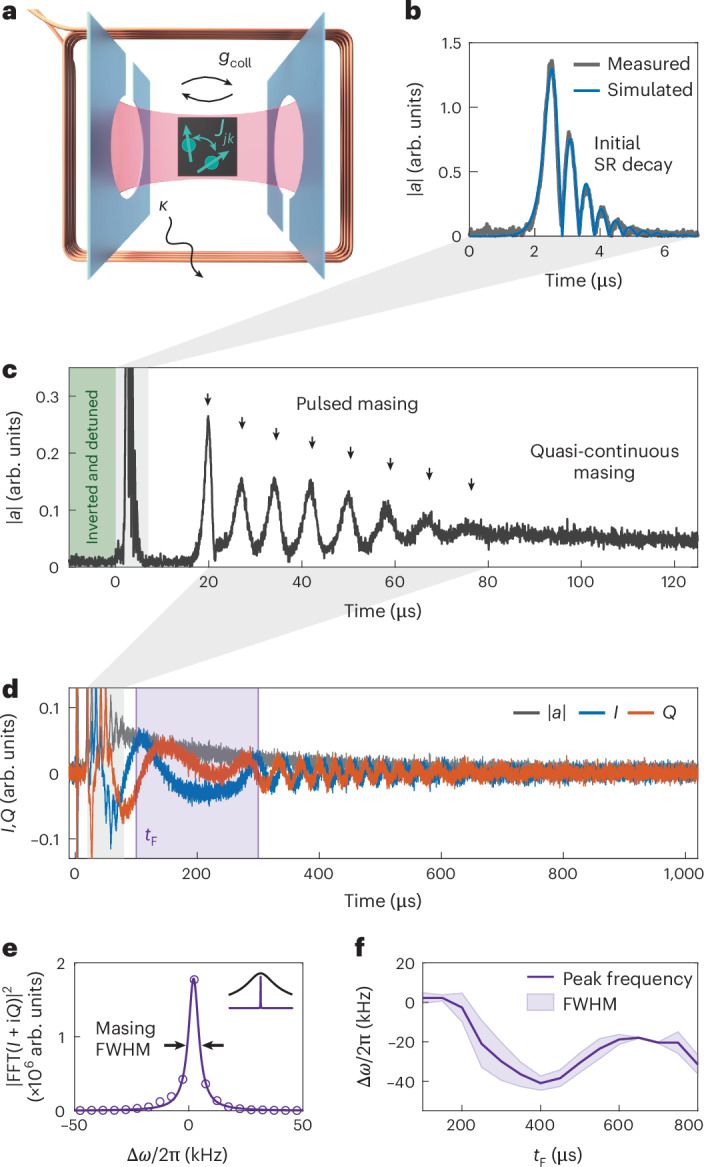
Experimental setup, self-induced superradiant masing dynamics and emission spectrum. **a**, Schematic of the superconducting microwave cavity strongly coupled to the NV^−^ diamond. **b**, Zoomed-in plot of the cavity amplitude ∣*a*∣ during the initial superradiant (SR) decay, which is triggered on tuning the inverted spin ensemble back into resonance and is well described by a semiclassical model. **c**, Expanding the time axis after the initial superradiant decay, we observe a series of narrow masing pulses evolving into a quasi-continuous cavity emission. Note the different *y*-axis scalings for ∣*a*∣ compared with that in **b**. **d**, Long tail of the quasi-continuous masing emission, showing the quadratures *I* and *Q* of the cavity amplitude, digitally demodulated in the rotating frame of the cavity resonance frequency for visual clarity. **e**, Fourier analysis (interval marked by the purple-shaded area in **d**), where the emission has a linewidth much smaller than the cavity (inset). The frequency difference Δ*ω*/2π is measured relative to the cavity frequency of 3.1 GHz. **f**, Frequency and linewidth of the emission change over time when the window of the Fourier analysis is shifted (see the main text for details). FFT, fast Fourier transform.

In our experiment, we investigate an inhomogeneously broadened ensemble of NV^−^ centres strongly coupled to a microwave cavity. Our system (Fig. 1a) allows for the generation of an inverted spin ensemble by applying strong microwave pulses, and for the controlled release of this inversion in the form of superradiant emission. The cavity, made of two parallel sapphire chips with superconducting split-ring structures, has a resonance frequency of around *ω*_c_/2π = 3.1 GHz. The roughly 200-μm-sized diamond sample is positioned between the chips. A wire loop wrapped directly around the chips is used for the rapid switching of the external magnetic field, allowing us to activate or suppress the cavity–spin interaction on demand. This assembly is housed in a copper box inside a dilution refrigerator, cooled to 25 mK and connected to a heterodyne detection scheme. The hybrid system of cavity and spin ensemble, with *N* ≈ 9 × 10^12^ NV^−^ centres, is well within the collective strong-coupling regime, having a cooperativity *C* = 14.6. Uniform cavity–spin coupling over the sample volume guarantees the required spin permutation symmetry to enable the superradiant dynamics we observe. In this dense spin ensemble, the typical nearest-neighbour distance between the NV^−^ spins is *r* = (*N*/*V*)^−1/3^ ≈ 8 nm. This results in a typical spin–spin coupling strength of about 100 kHz between neighbouring NV^−^s. Further details on the diamond sample, cavity and setup are provided in the Methods.

## Results

### Initial superradiant decay and spectral hole

For all experiments, we use the previously established protocol^34^ for generating a uniformly inverted spin state. All NV^−^ spins are tuned into resonance with the cavity, using a static magnetic field with equal projections along the four diamond axes. We apply a microwave inversion pulse to homogeneously invert all spins from a relaxed initial state of the effective two-level systems. Subsequently, we rapidly detune the spin ensemble from the cavity resonance and store the inversion for a set hold time. This procedure allows for the preparation of states with uniform initial spin inversion ${p}_{0}=\langle {\sigma }_{j}^{z}\rangle$ and almost-zero transversal spin components $\langle {\sigma }_{j}^{-}\rangle \approx 0$. The values for *p*_0_, bounded by ±1, are tunable within the range of 0.1–0.4 by modifying the hold time on the order of milliseconds.

On tuning the spins back into resonance with the cavity using the detuning loop, the inverted spin state is free to interact with the cavity mode. If the stored inversion exceeds the threshold *p*_0_*C* > 1, the system enters a metastable state^35^. Here $C={g}_{{\rm{coll}}}^{2}/\kappa \varGamma \approx 14.6$ is the cooperativity, a dimensionless parameter combining the collective coupling strength *g*_coll_/2π = 4.53 MHz, the cavity linewidth *κ*/2π = 418 kHz (half-width at half-maximum) and the effective ensemble dephasing rate *Γ*/2π = 3.36 MHz. The latter accounts for both inhomogeneously broadened spin frequency distribution *ρ*(*ω*), with a full-width at half-maximum (FWHM) of *W*/2π = 8.65 MHz, and the spin linewidths modelled by *γ*_⊥_/2π = 179 kHz (equations (9) and (10)).

In this metastable inverted state with *p*_0_*C* > 1, any fluctuation will stimulate a collective emission process known as a superradiant decay^36^. Conversely, if the inversion is below the instability threshold, dephasing due to inhomogeneous broadening becomes dominant and prevents this avalanche process^35^. In our case, the superradiant decay is triggered by noise photons from the input line. As the spin decay accelerates, the cavity amplitude ∣*a*∣ increases. It reaches its maximum as the collective spin vector points towards the equator of the Bloch sphere, where the cavity amplitude $\max (| a| )\propto {p}_{0}-1/C$ serves as a measure of the initial inversion above threshold (equation (12)), and the emitted intensity $| a{| }^{2} \approx {({p}_{0}N)}^{2}$ exhibits the characteristic quadratic scaling of superradiance with the number of effectively participating spins^2,34^. Subsequently, energy is coherently exchanged between cavity and spins, visible as damped Rabi oscillations (Fig. 1b). This coherent exchange is eventually stopped by coherence-limiting processes in the system, mainly the dephasing of inhomogeneously broadened spins.

Crucially, the cavity-resonant spins—which dominate the collective emission dynamics—become de-excited through superradiant emission, whereas the off-resonant spins maintain their inversion. This leaves behind a spectral hole^37^, a region of depleted inversion centred at the cavity frequency. This initial superradiant decay dynamics is well captured by the Maxwell–Bloch equations^38^ that provide a semiclassical description of the collective coupling between a non-interacting spin ensemble and the cavity mode. This standard picture does not predict further dynamics beyond the initial emission pulse, once the resonant spin inversion has been sufficiently depleted below the threshold *p*(*Δ* = 0) < 1/*C* (equation (11)).

### Pulsed and quasi-continuous masing

Surprisingly, however, we observe a train of masing pulses that emerges at about Δ*t* ≈ 15 μs after the initial superradiant burst (Fig. 1c). This behaviour cannot be understood within the standard semiclassical theory for emitter ensembles in cavities^38^ and suggests a dynamical refilling of the spectral hole burned by the collective cavity emission. The timescale for the revival pulses appears unexpectedly long, greatly exceeding the characteristic timescales of the cavity loss rate *κ*^−1^, the effective ensemble dephasing *Γ*^−1^, the collective cavity coupling ${g}_{{\rm{coll}}}^{\;-1}$ and the spin decoherence ${\gamma }_{\perp }^{-1}$. This clearly excludes Rabi oscillations or spin-echo effects as potential explanations^39^ for the observed superradiant pulse trains.

The masing pulses appear as distinct Gaussian-like peaks in the cavity amplitude with progressively increasing FWHM values ranging from around 1.5 μs to 3.8 μs. Each pulse has a random but nearly constant phase, as determined from the quadratures *I* and *Q* of the cavity amplitude, corresponding to coherent pulsed masing with near-transform-limited FWHM bandwidths ranging from around 400 kHz to 120 kHz. Following the early sequence of discrete pulses, the emission evolves into a quasi-continuous regime that persists for up to 1 ms.

The extended cavity dynamics (Fig. 1d) is measured using the same system parameters as those in Fig. 1c, but at half the digitizer sample rate in the heterodyne detection chain. The recorded quadratures *I* and *Q* of the cavity amplitude are demodulated at an intermediate frequency of 5 MHz detuned from the cavity resonance. We extract the emission’s linewidth by using fast Fourier transform analysis and Lorentzian profile fitting within the integration window of 200 μs (Fig. 1e). Shifting the start of the integration window *t*_F_, we analyse (Fig. 1f) how the central frequency of the masing emission drifts over time within ±25 kHz. The observed linewidth, varying from 5 kHz to 20 kHz, is two orders of magnitude below the cavity linewidth *κ* and the individual spin linewidth *γ*_⊥_, highlighting the crucial role of collective enhancement—an indicative trait of superradiance—in achieving high coherence. We attribute the linewidth variation to the emission frequency drift within the integration window caused by magnetic field oscillations after rapidly switching the detuning loop. These oscillations appear on a scale that is three orders of magnitude smaller than the full extent of the loop detuning of roughly 20 MHz.

### Experimental evidence for spectral hole refilling

**Fig. 2:**
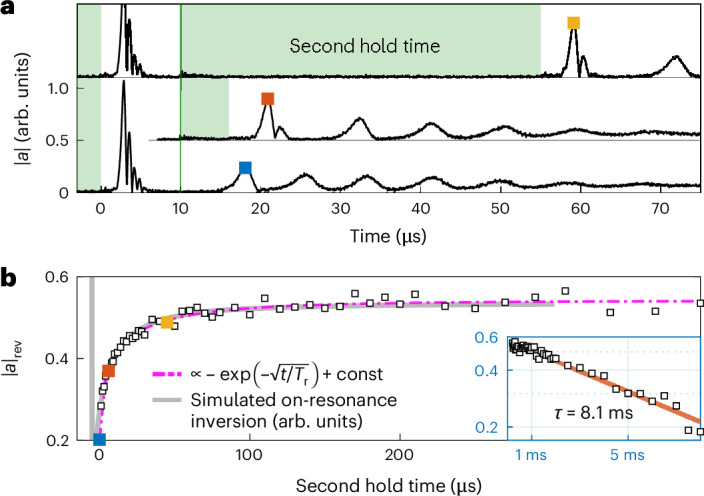
Influence of second hold time on revival dynamics. **a**, Stacked cavity signals of the superradiant dynamics with a second stabilization sequence (hold time), represented by the light-green shading. Increasing the duration of this second hold time extends the spectral hole-filling process, influencing the amplitude of the superradiant masing pulse revival. **b**, Revival amplitude ∣*a*∣_rev_ for varying second hold times. An initial stretched exponential increase is followed by an exponential decrease for longer timescales (inset). The data points are well described by simulating the on-resonance inversion using the parameters employed in Fig. 3.

To identify the mechanism responsible for the apparent spectral hole refilling, we conduct a set of auxiliary measurements designed to decouple the dynamics within the spin system from the cavity. Right after the initial superradiant decay, we rapidly detune the spins and introduce a second hold time, during which the spin–cavity interaction is suppressed. Extending this isolation phase reveals that the amplitude of the first revival pulse increases with longer off-resonant hold times (Fig. 2a,b). This strong dependence on the isolation period clearly establishes that the cavity coupling plays no substantial role in the spectral hole refilling, which instead must be driven by another additional mechanism. When the spins are tuned back into resonance, the accumulated inversion inside the hole—now above the instability threshold—triggers a superradiant pulse, whose amplitude scales with the amount of resonant spin inversion, which initially increases with the hold time. At longer hold times, once the spectral hole is maximally refilled, the amplitude reaches a plateau (Fig. 2b). The eventual decrease in amplitude over longer timescales reflects a global loss of spin inversion, as previously discussed in ref. ^34^.

**Extended Data Fig. 1:**
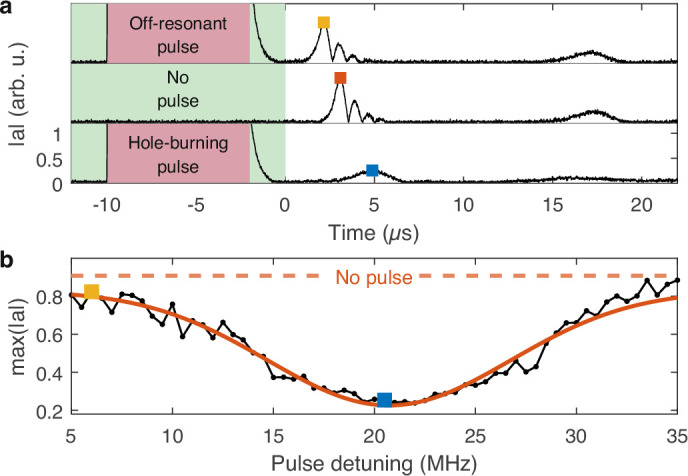
Probing superradiant dynamics after a hole-burning pulse. **a**, Cavity dynamics when applying a strong microwave pulse (red shading) to the spins while detuned (green shading) and subsequently triggering the superradiant decay and revival pulses. This will create a spectral hole in the ensemble, resulting in a slice of decreased spin inversion at varying frequencies for different runs. A resonant hole-burning pulse reproduces a spectral hole similar to the one created by the initial superradiant decay when no hole-burning pulse is applied. When the hole-burning pulse is off-resonant at the sides of the spin distribution, it still leads to an earlier triggering of the initial superradiant decay, but will otherwise recreate similar dynamics as when no pulse is applied. **b**, Maximum amplitude of the superradiant decay pulse for different hole-burning frequencies, revealing the frequency distribution of the detuned spins, although appearing with a larger width due to power broadening.

Additionally, in Extended Data Fig. 1, we closely reproduce the revival pulse dynamics by deliberately creating a spectral hole with a microwave pulse, confirming that the refilling mechanism operates independently of how the hole is initially formed. Although the observed pulsed masing bears resemblance to recently reported periodic superradiance in optically pumped spin systems^40^, our system operates in a fundamentally different regime: the pulses emerge spontaneously without any external pump, pointing to a self-induced mechanism originating within the spin ensemble.

### Many-body dynamics through dipole–dipole interactions in an NV^−^ ensemble

**Fig. 3:**
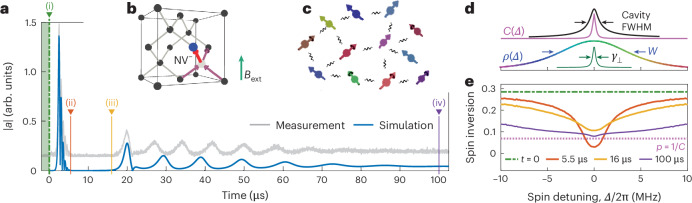
Simulation of superradiant dynamics driven by spin–spin interactions. **a**, Simulation of the cavity amplitude ∣*a*∣ based on the microscopic spin model compared with the measurement (offset vertically for clarity), with four key time steps marked (as shown in **e**). **b**, NV^−^ centre in a diamond unit cell, illustrating one of four possible alignments. **c**, Schematic of the simulated spin network: NV^−^ centres are randomly distributed in space, orientation and resonance frequency, and interact via dipole–dipole couplings. **d**, Relevant linewidths in the hybrid system: cavity linewidth, inhomogeneous spin distribution of width *W*, single-spin dephasing rate *γ*_⊥_ and frequency-dependent cooperativity function *C*(*Δ*). **e**, Simulated spin inversion profiles at four key times: (i) at *t* = 0, uniform inversion; (ii) at *t* = 5.5 μs, deep spectral hole; (iii) at *t* = 16 μs, refilled above threshold; (iv) at *t* = 100 μs, broad, depleted quasi-steady state.

To understand and explain our experimental observations, we have performed microscopic simulations of the cavity-coupled spin ensemble in the presence of direct interactions between the emitters. As detailed in Supplementary Sections 1 and 2, the magnetic dipole interactions between the NV^−^ centres generate a coherent exchange of spin excitations, which offers a possible mechanism for transporting inversion across the energy spectrum of the inhomogeneously broadened spin ensemble. The proper description of the collective spin–cavity coupling and the resulting superradiant emission dynamics requires sizable particle numbers to reliably sample the random positions, frequencies and orientations of the spins. Hence, we have implemented microscopic simulations of the resulting many-body dynamics for large numbers of ~10^6^ particles, with randomly sampled positions **r**_*j*_ in a cubic box to resemble our experimental NV^−^ density, random cavity detunings *Δ*_*j*_ drawn from the known experimental spectrum *ρ*(*Δ*) (Fig. 3d) and randomly chosen spin orientations from one of the four possible directions for the tetrahedral symmetry of diamond (Fig. 3b). Noting that the single-spin decoherence rate *γ*_⊥_ exceeds the typical strength *J*_*jk*_ of pairwise dipole–dipole interactions, one can derive an effective rate description for the interaction-driven evolution of the spin inversion as

1 $${\partial }_{t}{p}_{j}=-\sum _{k}\frac{4{\gamma }_{\perp }| {J}_{jk}{| }^{2}}{{({{\mathit\varDelta }}_{j}-{{\mathit\varDelta }}_{k})}^{2}+4{\gamma }_{\perp }^{2}}\left({p}_{j}-{p}_{k}\right),$$

which, in turn, can strongly affect the many-body emission dynamics due to the collective cavity coupling. Equation (1) describes the transport of spin excitations across the sample and across the energy spectrum of the spin ensemble with a two-body rate ∣*J*_*jk*_∣^2^ ∝ 1/∣**r**_*k*_ − **r**_*j*_∣^6^ that is only effective for closely adjacent NV^−^ centres.

In Fig. 3, we show the simulation results for the spin–cavity dynamics in the presence of dipole–dipole interactions. We infer a starting inversion of *p*_0_ = 0.285 from the initial superradiant decay and set the typical nearest-neighbour NV^−^ distance to *r* ≈ 7 nm to align the onset of pulsed masing with the experimental data, in good agreement with the independently estimated value of 8 nm based on the collective coupling strength and cavity mode volume (Methods).

The simulation quantitatively reproduces the dynamics up to the first revival pulse and qualitatively captures the key features of superradiant emission throughout the measurement interval, from the initial sequence of periodic pulses followed by the quasi-continuous masing regime. Although the precise spacing of the periodic pulses is not perfectly reproduced, the theory captures the characteristic shape of the masing pulses as well as their steadily increasing delay times.

**Extended Data Fig. 2:**
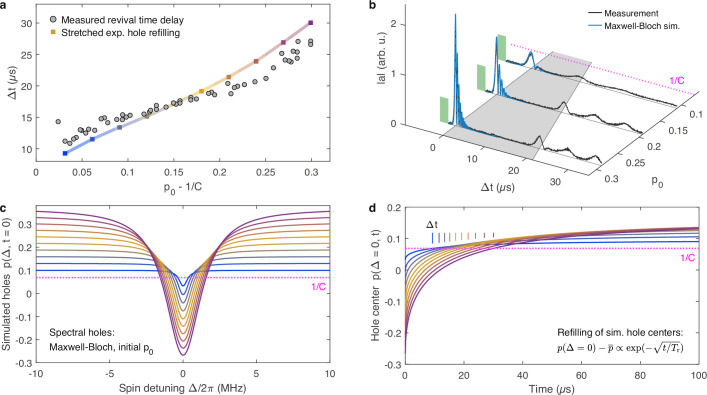
Influence of initial inversion on refilling time. **a**, Measured time delay *Δt* between the initial superradiant decay and the first revival pulse (circles), plotted as a function of the reduced initial inversion *p*_0_ − 1/*C*. This reduced inversion corresponds to the depth of the spectral hole created by the initial emission, since the resonant spins involved effectively undergo a *π*-rotation (cf. panel **c**). **b**, Experimental runs for three different initial inversion levels *p*_0_, illustrating how *Δt* is extracted from the cavity signal ∣*a*∣. The values of *p*_0_ are obtained by fitting the initial superradiant decay using Maxwell-Bloch equations [Eqs. (8c) without spin-spin interactions]. **c**, From such Maxwell-Bloch simulations, we obtain access to the inversion profile. These profiles are shown for different initial *p*_0_, evaluated at the time when the cavity amplitude ∣*a*∣ reaches its peak value during the initial superradiant pulse. In all cases, the initial inversion *p*_0_ is assumed to be uniform across the spin ensemble. **d**, Using the stretched exponential relaxation of the on-resonance inversion, Eq. (2), we can estimate the refilling dynamics with the experimentally obtained time constant *T*_r_ = 11.6 *μ*s from the fit of Fig. 2b. We extract from these curves the time *Δt* when the on-resonance inversion reaches 1/*C* (squares in panel **a**). This time delay *Δt* serves as a measure for how long it takes to refill the spectral hole above the instability threshold *p*(*Δ* = 0) > 1/*C*, enabling the revival pulse. We find good agreement between the experiment and the estimated time delay.

**Extended Data Fig. 3:**
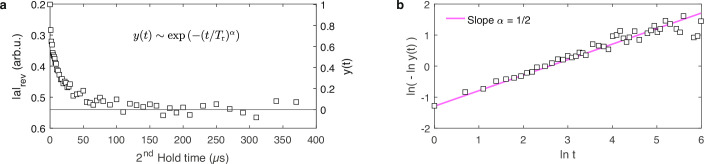
Stretching exponent evaluation of spectral hole refilling. **a**, Data from Fig. 2b, normalized to define the relaxation function *y*(*t*), which decays from 1 to 0 with a characteristic stretching exponent *α*. **b**, Double-logarithmic plot of ln (− ln*y*(*t*)) versus ln*t*. The linear trend confirms stretched exponential dynamics, and the slope yields a direct estimate of the stretching exponent *α* ≈ 1/2. This value is direct evidence of the expected 1/*r*^3^ scaling of dipolar spin-spin interactions in three dimensions; see Supplementary Information and ref. ^32^.

To further corroborate the important role of dipole–dipole interactions, we also studied the refilling of the spectral hole in the absence of spin–cavity coupling, as measured experimentally (Fig. 2). We use the fact that the amplitude of a superradiant pulse directly results from the cavity-resonant spin inversion just before pulse onset (equation (12)). The measurement of ∣*a*∣_rev_ (Fig. 2a), thus, provides direct access to the evolution of *p*(*Δ* = 0), and is shown as a function of the hold time (Fig. 2b). For comparison, we have simulated the spin dynamics, using the same parameters as employed in Fig. 3, but switching off the spin–cavity interaction after the initial superradiant decay. After accounting for a time offset between the initial decay and the first revival pulse, we find excellent agreement between experiment and theory, and observe that the hole refilling dynamics follow a stretched exponential:

2 $$p({\varDelta} =0)-{\overline{p}}\propto \exp (-\sqrt{t/{T}_{\rm{r}}}),$$

where $\overline{p}$ is the average inversion of the ensemble. Fitting the revival pulse amplitude as a function of the second hold time yields a characteristic time constant *T*_r_ ≈ 11.6 μs (Fig. 2b). This behaviour is consistent with an independent measurement of the refilling time, obtained from the time delay Δ*t* between the initial superradiant decay and the first revival pulse for different initial inversions *p*_0_ (Extended Data Fig. 2). The stretched exponential relaxation emerges from a simplified analytical solution to equation (1) (Supplementary Section 3). Importantly, the observed exponent of 1/2 (Extended Data Fig. 3) is a hallmark of the 1/*r*^3^ dependence of dipolar interactions^32^. We, thus, conclude that the dipole–dipole interactions between the spins are indeed responsible for the transfer of inversion with and without spin–cavity interactions.

On the basis of the above insights, we can now draw a conclusive picture of the observed superradiant masing dynamics. To this end, we show (Fig. 3e) the inversion profile at four key times during the measured and simulated dynamics. (i) All spins start fully inverted, well above the threshold. (ii) The initial superradiant decay generates a deep spectral hole around the cavity resonance, lowering the spin inversion below the threshold. Now, dipole–dipole interactions steadily repopulate the on-resonance spin inversion *p*(*Δ* = 0). (iii) The first revival pulse emerges after the refilled inversion crosses the instability threshold, triggered by residual cavity photons. Its amplitude reflects how much *p*(*Δ* = 0) has grown above the threshold. Following the simultaneous creation of the spectral hole, the refilling process starts anew. As more and more energy is emitted by the cavity, each subsequent pulse has fewer participating excited spins and, thus, generates a shallower hole, which leads to faster refilling. At the same time, the superradiant buildup gradually slows down, due to the 1/*p* scaling of the associated timescale^41^. As a result, the system transitions from an initial pulsed regime to a quasi-steady state (iv) in which energy is continuously replenished from the off-resonant spins, simultaneously being emitted through the cavity. This process eventually halts when the masing cannot be upheld due to considerable inversion loss.

## Discussion

We have demonstrated a novel manifestation of superradiant masing in a hybrid system composed of NV^−^ centre spins in a diamond coupled to a superconducting microwave cavity. Our observation of self-induced pulsed and quasi-continuous superradiant masing is well reproduced by a microscopic model that implements dipole–dipole interactions together with the coupling to the cavity. Remarkably, dipolar interactions, far from being merely a decoherence channel, result in a redistribution of energy that enables collective emission in a strongly disordered ensemble.

This behaviour highlights that dense dipolar spin networks, often idealized as formally closed many-body systems^42,43^, can effectively act as their own bath and relax towards thermal-like states on microsecond timescales. A finite *T*_2_ = 1/*γ*_⊥_ (combining intrinsic and background-spin-induced broadening) ensures sufficient spectral overlap and enforces equilibration. An open question is whether this self-thermalization—and the associated hole refilling—can be suppressed or modified by ensemble-splitting or dynamical-decoupling protocols, a subject for future exploration on our tunable NV^−^ platform.

Beyond fundamental insights, our findings suggest a route to a solid-state superradiant maser driven by spin–spin interactions. By repeatedly re-exciting off-resonant spins, one could sustain inversion without optical pumping^28^. This concept parallels recent diamond amplifiers that exploit cross-relaxation between NV^−^ centres and nitrogen impurities^44^, and may enable ultranarrow-linewidth microwave sources for precision metrology^31^.

## Methods

### Spin ensemble

**Extended Data Fig. 4:**
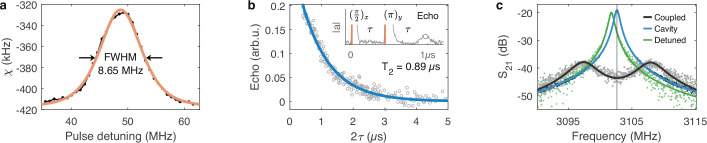
Spin distribution, *T*_2_, and steady-state spectroscopy measurements. **a**, Measurement of the inhomogeneously broadened spin distribution in the dispersive regime, with spins detuned by around *δ*/2*π* ≈ 50 MHz from the cavity. Following a 100 ms weak microwave drive at different frequencies, with the spin system initially in the ground state, only spins with the corresponding frequency will end up in an equal mixture of up and down spins, while other spins are unaffected. This change in the overall spin ensemble inversion leads to a dispersive shift *χ* ∝ 〈*S*_*z*_〉 of the cavity resonance frequency, measured using a vector network analyzer. **b**, Measurement of *T*_2_ using a Hahn echo sequence with ${(\frac{\pi }{2})}_{x}$ and (*π*)_*y*_ pulses of 20 ns duration. **c**, Transmission spectroscopy of the hybrid system using high (blue) and low (black) microwave power. Using high input power, the whole spin ensemble is brought into a completely mixed state, effectively decoupling it from the cavity. A Lorentzian fit yields a linewidth of *κ*/2*π* = 418 kHz. Using low input power, the normal-mode splitting of the coupled system in the ground state is measured and fitted with a collective coupling strength of *g*_coll_/2*π* = 4.53 MHz. When the spins are detuned (green), the response at low input power shows the suppression of cavity-spin interaction.

The spin system used in this work is the negatively charged NV^−^ centre in diamond. The ground-state Hamiltonian of the defect with spin *S* = 1 is given by ${\mathcal{H}}=\hslash D{S}_{z}^{2}+{\upmu} {\bf{B}}\cdot {\bf{S}}$, with the zero-field splitting *D*/2π = 2.88 GHz and μ/2π = 28 MHz mT^−1^. The diamond is cut from a larger sample with an initial nitrogen concentration of approximately 200 ppm and naturally abundant ^13^C isotopes, treated with neutron irradiation with a total fluence of 5 × 10^17^ cm^−1^ for 50 h for the creation of lattice vacancies, and subsequently annealed at 900 °C for 3 h for the formation of NV^−^ centres. The high amount of lattice damage due to neutron irradiation and the high spin concentration are the reasons for the large inhomogeneous broadening *W*/2π = 8.65 MHz and short *T*_2_ = 1/*γ*_⊥_ = 0.89 μs (Extended Data Fig. 4a,b).

### Hybrid system characterization

We measure the inhomogeneously broadened spin distribution in the dispersive regime (Extended Data Fig. 4a). This distribution is well described by a *q*-Gaussian function^45^:

3 $$\rho ({\varDelta} )=\frac{1}{{\mathcal{C}}}{\left[1-\left(1-q\right){{\varDelta }}^{2}/{\delta }_{q}^{2}\right]}^{\frac{1}{1-q}}\,,$$

with a normalization constant ${\mathcal{C}}$, ${\delta }_{q}=W\sqrt{(q-1)/({2}^{q-1}-1)}$ and *W* being the FWHM, using a value for the shape parameter of *q* = 1.39. We determine the cavity linewidth *κ*/2π = 418 kHz by measuring the steady-state transmission of the coupled system with a high input power of the vector network analyser (Extended Data Fig. 4c). This brings the whole spin ensemble into a completely mixed state, effectively decoupling it from the cavity. Using low input power, we measure the normal-mode splitting of the system in the ground state. A steady-state solution of equation (8c) without dipole–dipole interactions provides the fitted value of *g*_coll_/2π = 4.53 MHz. Comparing this value with the single spin coupling *g*_0_/2π ≈ 1.5 Hz (obtained from a finite-element simulation of the cavity), we get an estimation of the number of spins $N={g}_{{\rm{coll}}}^{2}/{g}_{0}^{2}\approx 9\times 1{0}^{12}$. Taking the volume of the diamond sample of around 5 × 10^6^ μm^3^ and the carbon density in diamond of *n*_c_ = 1.755 × 10^23^ cm^−3^ into account results in an NV^−^ concentration of approximately 10 ppm and a typical nearest-neighbour distance between the NV^−^s of *r* = (*N*/*V*)^−1/3^ ≈ 8 nm.

### Microwave setup, cavity and inversion pulse

We use *I*/*Q* mixing with an arbitrary waveform generator and a microwave source at the cavity frequency to synthesize the inversion pulse. After amplification with a high-power amplifier, the pulse is injected into cavity port 1. The outgoing signal, exiting at cavity port 2, undergoes further amplification both inside and outside the cryostat. It is demodulated using another frequency source and recorded by a digitizer with a maximum sampling rate of 2 GHz. Additional details on the superconducting cavity design and the protocol for initializing the inverted state, including details of the inversion pulse and detuning loop switching, are available in ref. ^34^.

### Dipole–dipole interactions and spectral hole refilling

To describe the refilling of depleted resonant spins, we consider the dipole–dipole interaction Hamiltonian

4 $${{\mathcal{H}}}_{{\rm{dipole}}}=-\hslash \sum\limits_{j,k\ > \ j}\,\frac{{J}_{0}}{| {{\bf{r}}}_{jk}{| }^{3}}\frac{1}{{\hslash }^{2}}\left[3({{\bf{S}}}_{j}\cdot {\hat{{\bf{u}}}}_{jk})({{\bf{S}}}_{k}\cdot {\hat{{\bf{u}}}}_{jk})-{{\bf{S}}}_{j}\cdot {{\bf{S}}}_{k}\right],$$

where *J*_0_/2π = 51.9 MHz nm^3^, **r**_*jk*_ is the vector connecting the spins and ${\hat{{\bf{u}}}}_{jk}={{\bf{r}}}_{jk}/| {{\bf{r}}}_{jk}|$. In our experiment, only the transition between the *m* = 0 and *m* = +1 magnetic sublevels is of relevance. Taking into account the four different orientations of NV^−^s in our system, we can write the Hamiltonian as

5 $${{\mathcal{H}}}_{{\rm{dd}}}=\hslash{\sum}_{{j,k}\atop{k> j}}\left[\left({J}_{jk}{\sigma }_{j}^{+}{\sigma }_{k}^{-}+{J}_{jk}^{* }{\sigma }_{k}^{+}{\sigma }_{j}^{-}\right)+{Q}_{jk}{\sigma }_{j}^{\;\rm{ee}}{\sigma }_{k}^{\;\rm{ee}}\right],$$

6 $${\rm{with}}\quad {J}_{jk}=-\frac{{J}_{0}}{| {r}_{jk}{| }^{3}}({g}_{jk}+{\rm{i}}{h}_{jk}),\quad {Q}_{jk}=-\frac{{J}_{0}}{| {r}_{jk}{| }^{3}},$$

where $${g}_{jk}=\frac{1}{2}({T}_{jk}^{\;xx}+{T}_{jk}^{yy})$$, $${h}_{jk}=\frac{1}{2}({T}_{jk}^{xy}-{T}_{jk}^{yx})$$ and $${q}_{jk}={T}_{jk}^{zz}$$, with $${T}_{jk}^{\alpha \beta }$$$$=3({\hat{{\bf{e}}}}_{{O}_{j}}^{\alpha }\cdot {\hat{{\bf{u}}}}_{jk})({\hat{{\bf{e}}}}_{{O}_{k}}^{\beta }\cdot {\hat{{\bf{u}}}}_{jk})-{\hat{{\bf{e}}}}_{{O}_{j}}^{\alpha }\cdot {\hat{{\bf{e}}}}_{{O}_{k}}^{\beta }$$. Here $${\hat{{\bf{e}}}}_{{O}_{j}}^{\alpha }$$ denotes the *α*-oriented unit vector of the local coordinate system at site *j*, with *α*, *β* ∈ {*x*, *y*, *z*} (Supplementary Section 1).

The total Hamiltonian is given by

7 $${\mathcal{H}}=\hslash \sum _{j}{{\mathit\varDelta} }_{j}{\sigma }_{j}^{\rm{ee}}+\hslash {g}_{0}\sum _{j}\left({a}^{\dagger }{\sigma }_{j}^{-}+{\sigma }_{j}^{+}a\right)+{\rm{i}}\hslash \eta \left({a}^{\dagger }-a\right)+{{\mathcal{H}}}_{{\rm{dd}}},$$

with *a*^†^ (*a*) being the creation (annihilation) operator of the cavity mode and ${\sigma }_{j}^{\rm{ee}},{\sigma }_{j}^{\pm }$ being the projection on the excited state and raising/lowering operators for the *j*th spin, respectively. The cavity losses are accounted for by a rate *κ*. We disregard *T*_1_ processes as the spin-lattice relaxation rate is negligible compared with all other dynamical timescales of our system. In our simulations, we also consider a weak external driving field *η* to model the triggering of superradiant pulses by technical noise. It mainly affects the precise timing of the first revival pulse as well as its shape.

We neglect three-spin and cavity-induced contributions in the dynamics of the spin–spin coherences, $\langle {\sigma }_{j}^{+}{\sigma }_{k}^{-}\rangle$, as they have negligible impact on the dynamics. Since the (single-spin) decoherence rate *γ*_⊥_ is the dominant timescale, we adiabatically eliminate the dynamics of these correlations, ${\partial }_{t}\langle {\sigma }_{j}^{+}{\sigma }_{k}^{-}\rangle \approx 0$. Factorizing spin–cavity, $\langle a{\sigma }_{j}^{+}\rangle \approx \langle a\rangle \,\langle {\sigma }_{j}^{+}\rangle$, and two-spin expectation values, $\langle {\sigma }_{j}^{\;{\rm{ee}}}{\sigma }_{k}^{-}\rangle \approx \langle {\sigma }_{j}^{\;\rm{ee}}\rangle \,\langle {\sigma }_{k}^{-}\rangle$, we arrive at the dynamical equations of our system:

8a $${\partial }_{t}\langle a\rangle =-\kappa \langle a\rangle -{\rm{i}}{g}_{0}\sum _{j}\langle {\sigma }_{j}^{-}\rangle +\eta \,,$$

8b $$\begin{array}{ll}{\partial }_{t}\langle {\sigma }_{j}^{-}\rangle=-({\gamma }_{\perp }+{\rm{i}}{{\varDelta} }_{j})\langle {\sigma }_{j}^{-}\rangle +{\rm{i}}{g}_{0}\langle a\rangle {p}_{j}\\\qquad\qquad+\,{\rm{i}}{p}_{j}\mathop{\sum}\limits_{{k}\atop{k\,\ne\,j}}{J}_{jk}\langle {\sigma }_{k}^{-}\rangle -{\rm{i}}\langle {\sigma }_{j}^{-}\rangle\mathop{\sum}\limits_{{k}\atop{k\,\ne\,j}}{Q}_{jk}({p}_{k}+1)/2\,,\end{array}$$

8c $${\partial}_{t}{p}_{j}=-4{g}_{0}{\rm{Im}}(\langle {a}^{\dagger}\rangle \langle {\sigma }_{j}^{-}\rangle )-\mathop{\sum}\limits_{{k}\atop{k\,\ne\,j}}\frac{4{\gamma }_{\perp }\vert {J}_{jk}{\vert}^{2}\left({p}_{j}-{p}_{k}\right)}{{({{\varDelta} }_{j}-{{\varDelta} }_{k})}^{2}+4{\gamma }_{\perp}^{2}}\,.$$

We sample ${n}_{{\rm{sim}}}=1{0}^{6}$ NV^−^ centres distributed randomly in a cube. For this number, we find convergence for the cavity amplitude with respect to the spatial and frequency distributions as well as the orientations of the NV^−^s. To take into account the actual number *N* of NV^−^s in the experiment, we have to consider $N/{n}_{{\rm{sim}}}$ copies of our box when coupling to the cavity in equation (8a). To avoid stiffness issues, we single out neighbouring spins that equilibrate faster than all other timescales in the system and approximate them to instantaneously equilibrate. We simulate the remaining equations using an explicit Runge–Kutta method. A detailed derivation is provided in Supplementary Section 3.

### Superradiant instability threshold and cooperativity

The instability criterion *p*_0_*C* > 1 is formally derived^35^ from equation (8c) as a necessary condition for the growth of the cavity amplitude starting from zero photons 〈*a*〉 = 0, in the case of uniform initial inversion ${p}_{0}=\langle {\sigma }_{j}^{z}\rangle$ and vanishing $\langle {\sigma }_{j}^{-}\rangle =0$, assuming no spin–spin interactions (*J*_*jk*_ = *Q*_*jk*_ = 0). The frequency-resolved single-spin cooperativity is given by

9 $$C({\varDelta})=\frac{{g}_{0}^{2}}{\kappa}{\left({\gamma}_{\perp}+\frac{{\varDelta}^{2}}{{\gamma}_{\perp}}\right)}^{-1}\,,$$

and defines the total cooperativity as an integral over the spin distribution (∫*ρ*(*Δ*) *dΔ* = *N*):

10 $$C=\,\int\,C({\varDelta} )\,\rho ({\varDelta} )\,d{{\varDelta}} =\frac{{g}_{{\rm{coll}}}^{2}}{\kappa {\varGamma}}\,,$$

where *Γ* is the effective ensemble linewidth, incorporating both inhomogeneous broadening and intrinsic spin dephasing. For our system, we find *C* = 14.6 and *Γ*/2π = 3.36 MHz. For a non-uniform inversion profile *p*(*Δ*), this criterion generalizes in the continuum limit as a weighted integral over spin detunings. The system becomes unstable when

11 $${\overline{pC}}=\,\int\,p({\varDelta} )\,C({\varDelta} )\,\rho ({\varDelta} )\,d{\varDelta} > 1\,.$$

This expression highlights that the instability is dominated by the spin inversion near resonance *p*(*Δ* = 0), where *C*(*Δ*) is sharply peaked. Although this threshold strictly applies only in the absence of spin–spin interactions, it remains a valuable heuristic for interpreting the self-pulsing behaviour.

Accordingly, the peak cavity amplitude during superradiant emission follows the relation

12 $$\max (| a| )\propto {p}_{0}-\frac{1}{C}\,$$

as a consequence of the equations of motion (equation (8c)). To motivate this relation, we consider the moment when the cavity field reaches its maximum: ∂_*t*_〈*a*〉 ≈ 0. Equation (8a) then implies $\langle a\rangle \propto {\sum }_{j}\langle {\sigma }_{j}^{-}\rangle \equiv {S}_{-}$. The transverse spin component *S*_−_ = *S*_*x*_ − i*S*_*y*_ is built up during the superradiant burst via the collective rotation of an initially +*z*-oriented ensemble and peaks simultaneously with the cavity amplitude in our superradiant regime. The system starts with *S*_*z*_ ∝ *p*_0_*N* and negligible initial coherence *S*_−_ ≈ 0, the latter maintained at almost all times by rapid single-spin dephasing *γ*_⊥_. Only during the superradiant emission does coherence buildup dynamically. This leads to a linear scaling $\max (| a| ) \approx {p}_{0}N$. The offset in equation (12) reflects the superradiant threshold: for *p*_0_ < 1/*C*, the system remains stable and does not emit collectively. Since the dynamics are dominated by cavity-resonant spins, this scaling primarily reflects the on-resonance inversion *p*(*Δ* = 0).

## Online content

Any methods, additional references, Nature Portfolio reporting summaries, source data, extended data, supplementary information, acknowledgements, peer review information; details of author contributions and competing interests; and statements of data and code availability are available at 10.1038/s41567-025-03123-0.

## Supplementary information

Supplementary Information Supplementary Sections 1–3.

## Data Availability

The datasets generated and analysed during the current study are available via Zenodo at 10.5281/zenodo.17406420 (ref. ^46^).

## Code availability

The MATLAB and Python scripts used for the data analysis and simulations are available via Zenodo at 10.5281/zenodo.17406420 (ref. ^46^).
