# Supplementary material for: Self-induced superradiant masing
Source: Nat Phys. 2026 Jan 2;22(1):158–63. doi: 10.1038/s41567-025-03123-0 (PMC12811124; doi:10.1038/s41567-025-03123-0)
Supplement: Supplementary file 1 — Supplementary Sections 1–3. [file 41567_2025_3123_MOESM1_ESM.pdf]

nature physics

Supplementary information https://doi.org/10.1038/s41567-025-03123-0

Self-induced superradiant masing

In the format provided by the authors and unedited

Contents

S1. Dipole-dipole interactions for different NV^- orientations 2

S2. Mean field dynamics 4

S3. Numerical simulation 5

Qualitative solution to the refilling dynamics 5

References 7

S1. Dipole-dipole interactions for different NV$^-$ orientations

In our microscopic description of the superradiant spin-cavity dynamics, we take into account the known form of the interaction between the magnetic dipoles of the NV$^-$ centres. Each dipole is oriented along one of the four directions defined by the diamond crystal, as illustrated in Fig. S1a. For each of the N considered NV$^-$s, we choose an axis $\hat{\mathbf{e}}^z_{O_k}$ that is aligned with one of these four directions, where the index O_k labels the orientation, i.e., it takes on a random value out of $O_k \in \{1, 2, 3, 4\}$ for each of the $k = 1, \ldots, N$ nitrogen-vacancy centres. Correspondingly, we choose the remaining two cartesian axes $\hat{\mathbf{e}}^x_{O_k}$ and $\hat{\mathbf{e}}^y_{O_k}$ such that $\{\hat{\mathbf{e}}^x_{O_k}, \hat{\mathbf{e}}^y_{O_k}, \hat{\mathbf{e}}^z_{O_k}\}$ forms a right-handed orthogonal system. In this way, we can define the spin operators

$$\mathbf{S}_k = \hat{\mathbf{e}}^x_{O_k} s^x_k + \hat{\mathbf{e}}^y_{O_k} s^y_k + \hat{\mathbf{e}}^z_{O_k} s^z_k, \tag{S1}$$

in the local coordinate systems aligned with the individual axis of each NV$^-$-centre in terms of the corresponding spin-1 operators

$$s^x_k = \frac{\hbar}{\sqrt{2}}(|0\rangle_k\langle -| + |+\rangle_k\langle 0| + |-\rangle_k\langle 0| + |0\rangle_k\langle +|), \tag{S2}$$

$$s^y_k = \frac{\hbar}{\sqrt{2}\mathrm{i}}(|0\rangle_k\langle -| + |+\rangle_k\langle 0| - |-\rangle_k\langle 0| - |0\rangle_k\langle +|), \tag{S3}$$

$$s^z_k = \hbar\left(|+\rangle_k\langle +| - |-\rangle_k\langle -|\right), \tag{S4}$$

in the global laboratory frame, for which the z-axis is defined by the direction of the applied magnetic field which is chosen to coincide with the orientation of the diamond unit cell as shown in Fig. S1a. The energy eigenvalues of the NV$^-$s are dominated by the zero-field splitting of about 2.88 GHz, such that we can approximate their eigenvectors as those of the local S^z_k operators. In addition, the applied magnetic field, $\mathbf{B}$, causes a linear Zeeman shift determined by the field projection $\hat{\mathbf{e}}^z_{O_k} \cdot \mathbf{B}$ onto the local z-axis of each NV$^-$ centre. In our experiments, the magnetic field strength is adjusted such that the resulting energy of one of the S^z_k sublevels is close to the cavity frequency of 3.1 GHz. We define this level to be the $m = 1$ state and can correspondingly neglect the $m = -1$ state, as its energy is far off resonance from the cavity. The relevant dynamics can thus be described in terms of effective spin-1/2 systems with respective transition and projection operators

$$\sigma^-_k = |0\rangle_k\langle +|, \tag{S5}$$

$$\sigma^+_k = |+\rangle_k\langle 0|, \tag{S6}$$

$$\sigma^{ee}_k = |+\rangle_k\langle +|. \tag{S7}$$

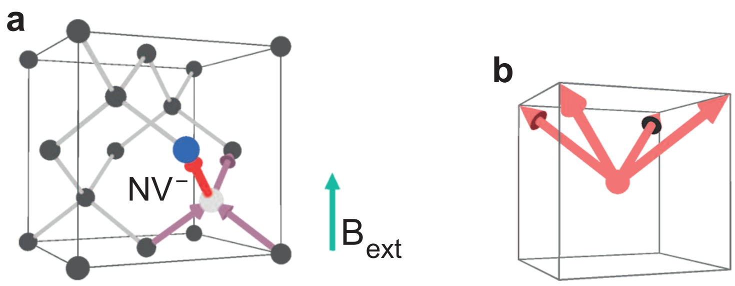

Supplementary Figure S1. **Orientations of the NV⁻ spins. a,** Schematic of an NV⁻ centre within the diamond unit cell, showing one of the four possible crystallographic orientations defined by the axis connecting the substitutional nitrogen atom (blue) to the adjacent lattice vacancy (light gray). **b,** Quantisation axes used in the microscopic theory. As a result of the external magnetic field, the tetrahedral symmetry of the four NV⁻ orientations is broken, and the axes form the edges of an inverted square pyramid.

This is equivalent to choosing the local coordinate systems such that $\hat{\mathbf{e}}^z_{O_k} \cdot \mathbf{B}$ is positive for all four orientations O_k, the $m = 1$ states are resonant with the cavity regardless of O_k. Specifically, this corresponds to (see Fig. S1b)

$$\hat{\mathbf{e}}^z_{O_k} \in \frac{1}{\sqrt{3}} \left\{ \begin{pmatrix} 1 \\ 1 \\ 1 \end{pmatrix}, \begin{pmatrix} 1 \\ -1 \\ 1 \end{pmatrix}, \begin{pmatrix} -1 \\ 1 \\ 1 \end{pmatrix}, \begin{pmatrix} -1 \\ -1 \\ 1 \end{pmatrix} \right\}. \tag{S8}$$

The remaining axes can then be constructed as cross products of $\hat{\mathbf{e}}^x_{O_k}$ and the global z-axis, $\hat{\mathbf{Z}} = (0, 0, 1)$,

$$\hat{\mathbf{e}}^x_{O_k} = \frac{\hat{\mathbf{Z}} \times \hat{\mathbf{e}}^z_{O_k}}{|\hat{\mathbf{Z}} \times \hat{\mathbf{e}}^z_{O_k}|}, \quad \hat{\mathbf{e}}^y_{O_k} = \hat{\mathbf{e}}^z_{O_k} \times \hat{\mathbf{e}}^x_{O_k}. \tag{S9}$$

The magnetic dipole-dipole interaction between the NV⁻ centres is described by the Hamiltonian

$$H_{\mathrm{dd}} = -\hbar \sum_{\substack{k,l \\ k \neq l}} \frac{\mu_0 \gamma_{\mathrm{e}}^2 \hbar}{4\pi |\mathbf{r}_{kl}|^3} \frac{1}{\hbar^2} \left[3(\mathbf{S}_k \cdot \hat{\mathbf{u}}_{kl})(\mathbf{S}_l \cdot \hat{\mathbf{u}}_{kl}) - \mathbf{S}_k \cdot \mathbf{S}_l \right] , \tag{S10}$$

where, μ_0 is the vacuum permeability, γ_{e} is the gyromagnetic ratio of the electron, and the unit vector $\hat{\mathbf{u}}_{kl} = \mathbf{r}_{kl}/r_{kl}$. We define the coupling constant

$$J_0 = \frac{\mu_0 \gamma_{\mathrm{e}}^2 \hbar}{4\pi} = 2\pi \times \frac{\mu_0 g_{\mathrm{e}}^2 \mu_{\mathrm{B}}^2}{8\pi^2 \hbar} = 2\pi \times 51.9\,\mathrm{MHz\,nm}^3. \tag{S11}$$

Substituting the local spin operators into the interaction Hamiltonian and neglecting counterrotating terms as well as transitions to the $m = -1$ level, as described above, yields the following effective Hamiltonian

$$H_{\mathrm{dd}} = \hbar \sum_{\substack{k,l \\ k < l}} \left[\left(J_{kl} \sigma_k^+ \sigma_l^- + J_{kl}^* \sigma_l^+ \sigma_k^- \right) + Q_{kl} \sigma_k^{ee} \sigma_l^{ee} \right] \tag{S12}$$

that describes the interaction between the spin-1/2 systems introduced in Eq.(S5). The coupling constants

$$J_{kl} = -\frac{J_0}{|r_{kl}|^3}(g_{kl} + \mathrm{i}h_{kl}), \qquad Q_{kl} = -\frac{J_0}{|r_{kl}|^3}q_{kl}. \tag{S13}$$

are anisotropic with an angular dependence that is given by

$$\begin{aligned} g_{kl} &= \frac{1}{2}(T_{kl}^{xx} + T_{kl}^{yy}), \quad h_{kl} = \frac{1}{2}(T_{kl}^{xy} - T_{kl}^{yx}), \quad q_{kl} = T_{kl}^{zz}, \\ T_{kl}^{\alpha\beta} &= 3(\hat{\mathbf{e}}_{O_k}^{\alpha} \cdot \hat{\mathbf{u}}_{kl})(\hat{\mathbf{e}}_{O_l}^{\beta} \cdot \hat{\mathbf{u}}_{kl}) - \hat{\mathbf{e}}_{O_k}^{\alpha} \cdot \hat{\mathbf{e}}_{O_l}^{\beta}, \end{aligned} \tag{S14}$$

where $\alpha, \beta \in \{x, y, z\}$.

S2. Mean field dynamics

The total Hamiltonian of our system can be written as

$$\mathcal{H} = \hbar \sum_i \Delta_i \sigma_i^{ee} + \hbar g_0 \sum_i \left(a^\dagger \sigma_i^- + \sigma_i^+ a\right) + i\hbar\eta \left(a^\dagger - a\right) + \mathcal{H}_{\mathrm{dd}}, \tag{S15}$$

with the spin-cavity detuning Δ, the (individual) spin-cavity coupling strength g_0, cavity operators $a, a^\dagger$ and cavity drive η. In addition to the coherent dynamics by the Hamiltonian, we also consider the individual dephasing $\gamma_\perp$ as well as the cavity loss κ in a Lindblad master equation, while we neglect T_1 processes. The relevant equations read

$$\partial_t \langle a \rangle = -\kappa \langle a \rangle - \mathrm{i}g_0 \sum_j \langle \sigma_j^- \rangle + \eta, \tag{S16}$$

$$\partial_t \langle \sigma_j^- \rangle = -(\gamma_\perp + \mathrm{i}\Delta_j)\langle \sigma_j^- \rangle + \mathrm{i}g_0 \langle a\sigma_j^z \rangle + \mathrm{i} \sum_{\substack{k \\ k \neq j}} J_{jk} \langle \sigma_j^z \sigma_k^- \rangle - \mathrm{i} \sum_{\substack{k \\ k \neq j}} Q_{jk} \langle \sigma_j^- \sigma_k^{ee} \rangle, \tag{S17}$$

$$\begin{aligned} \partial_t \langle \sigma_i^+ \sigma_j^- \rangle = &-[\mathrm{i}(\Delta_j - \Delta_i) + 2\gamma_\perp]\langle \sigma_i^+ \sigma_j^- \rangle + \mathrm{i}g_0(\langle a\sigma_i^+ \sigma_j^z \rangle - \langle a^\dagger \sigma_i^z \sigma_j^- \rangle) - \mathrm{i}J_{ji}(\langle \sigma_i^{ee} \rangle - \langle \sigma_j^{ee} \rangle) \\ &+ \mathrm{i} \sum_{\substack{l \\ l \neq i,j}} (J_{jl} \langle \sigma_i^+ \sigma_j^z \sigma_l^- \rangle - J_{il}^* \left\langle \sigma_l^+ \sigma_i^z \sigma_j^- \right\rangle) - \mathrm{i} \sum_{\substack{k \\ k \neq i,j}} (Q_{kj} \langle \sigma_i^+ \sigma_k^{ee} \sigma_j^- \rangle - Q_{ki} \langle \sigma_i^+ \sigma_k^{ee} \sigma_j^- \rangle) \end{aligned} \tag{S18}$$

$$\partial_t n_j = -2g_0 \,\mathrm{Im}(\langle a^\dagger \sigma_j^- \rangle) - \mathrm{i} \sum_{\substack{k \\ k \neq j}} \left(J_{jk} \langle \sigma_j^+ \sigma_k^- \rangle - J_{kj} \langle \sigma_k^+ \sigma_j^- \rangle \right), \tag{S19}$$

where we defined $n_j = \left\langle \sigma_j^{ee} \right\rangle$. The second line of Eq. (S18) contains terms that involve products of three spin operators. We numerically exclude their relevance on the refilling dynamics and neglect them in the following. We further neglect the cavity contributions to this equation because their effect on the refilling is suppressed with g_0^2/κ. Since $\gamma_\perp$ is the dominant timescale in the dynamics,

we can now adiabatically eliminate $\langle\sigma_i^+\sigma_j^-\rangle$ and reinsert the expression into Eq. (S19). Finally, we factorize spin-cavity, $\langle a\sigma_j^+\rangle \approx \langle a\rangle\langle\sigma_j^+\rangle$, and two-spin expectation values, $\langle\sigma_j^{ee}\sigma_i^-\rangle \approx \langle\sigma_j^{ee}\rangle\langle\sigma_i^-\rangle$ and arrive at the dynamical equations

$$\partial_t\langle a\rangle = -\kappa\langle a\rangle - \mathrm{i}g_0\sum_j\langle\sigma_j^-\rangle + \eta, \tag{S20a}$$

$$\partial_t\langle\sigma_j^-\rangle = -(\gamma_\perp + \mathrm{i}\Delta_j)\langle\sigma_j^-\rangle + \mathrm{i}g_0\langle a\rangle(2n_j-1) + \mathrm{i}(2n_j-1)\sum_{\substack{k\\k\neq j}} J_{jk}\,\langle\sigma_k^-\rangle - \mathrm{i}\,\langle\sigma_j^-\rangle\sum_{\substack{k\\k\neq j}} Q_{jk}n_k\ , \tag{S20b}$$

$$\partial_t n_j = -2g_0\,\mathrm{Im}(\langle a^\dagger\rangle\langle\sigma_j^-\rangle) - \sum_{\substack{k\\k\neq j}}\frac{4\gamma_\perp|J_{jk}|^2}{(\Delta_j-\Delta_k)^2+4\gamma_\perp^2}\,(n_j-n_k)\ . \tag{S20c}$$

S3. Numerical simulation

In our simulations, we consider up to $n_{\mathrm{sim}} = 10^6$ spins, thereby ensuring statistical significance when drawing from the distributions. We sample the spin frequencies from the experimentally determined distribution and consider the spins randomly distributed in a cube of appropriate size. In addition, we randomly sample the orientations of the NV^- centres. To correctly capture the spin-cavity interaction, we consider N/n_{sim} copies of the cube in the relevant sums of the above equations. We simulate these equations using an explicit Runge-Kutta method, which is suitable for the spin-cavity dynamics. The dipole-dipole-induced relaxation process introduces stiffness issues in the simulation. We tackle these issues by singling out strongly interacting neighbours and treating them as instantaneously relaxed, and project the equations on the corresponding reduced subspace.

Qualitative solution to the refilling dynamics

While the full numerical solution is available, it is instructive to consider an approximated scenario using the so-called *relaxation time approximation* to derive an analytical solution to the refilling dynamics. Note that the rates we derive within the approximation are far beyond the rates observed experimentally. Still, qualitative insights applicable to the scenario in the main text may be extracted from the model. We focus on the case of refilling in the absence of the cavity. Within the relaxation time approximation, we approximate that for each depopulated spin i all its neighbours k have already reached equilibrium, $n_k \approx n_{\mathrm{avg}}$, yielding the dynamical equations

$$\partial_t\Delta n_i = -\sum_{\substack{k\\k\neq i}}\frac{4\gamma|J_{ki}|^2}{(\Delta_k-\Delta_i)^2+4\gamma^2}\Delta n_i, \tag{S21}$$

where we defined $\Delta n_i = n_i - n_{\rm avg}$. This decouples the equations for different Δn_i, such that we can write the solution

$$\Delta n_i(t) = \exp\left(-\sum_{\substack{k\\k\neq i}} \frac{4\gamma|J_{ki}|^2 t}{(\Delta_k-\Delta_i)^2+4\gamma^2}\right)\Delta n_i(0). \tag{S22}$$

We can now perform the ensemble average on this solution regarding the detuning Δ, position $\vec{r}$ and the orientations O,

$$\begin{aligned}
&\left\langle \exp\left(-\sum_{\substack{k\\k\neq i}} \frac{4\gamma|J_{ki}|^2 t}{(\Delta_k-\Delta_i)^2+4\gamma^2}\right)\right\rangle = \\
&= \int \mathrm{d}\Delta_1 \dots \mathrm{d}\Delta_{N-1}\, \bar{n}(\Delta_1)\dots\bar{n}(\Delta_{N-1}) \int \frac{\mathrm{d}\vec{r}_1}{V}\dots\frac{\mathrm{d}\vec{r}_{N-1}}{V}\frac{1}{4}\sum_{O_1}\dots\frac{1}{4}\sum_{O_{N-1}} \times \\
&\qquad\qquad \times \exp\left(-\sum_{\substack{k\\k\neq i}} \frac{4\gamma|J_{ki}|^2 t}{(\Delta_k-\Delta_i)^2+4\gamma^2}\right) = \\
&= \left[\int \mathrm{d}\Delta_k\, \bar{n}(\Delta_k) \int \frac{\mathrm{d}\vec{r}}{V}\frac{1}{4}\sum_{O_k} \exp\left(-\frac{4\gamma t[g_{ki}^2+h_{ki}^2]}{(\Delta_k-\Delta_i)^2+4\gamma^2}\frac{J_0^2}{r^6}\right)\right]^{N-1} \\
&= \left[1-\frac{1}{N-1}\int \mathrm{d}\Delta_k\, \bar{n}(\Delta_k)\frac{1}{4}\sum_{O_k}\frac{N-1}{V}\int \mathrm{d}\vec{r}\left[1-\exp\left(-\frac{4\gamma t[g_{ki}^2+h_{ki}^2]}{(\Delta_k-\Delta_i)^2+4\gamma^2}\frac{J_0^2}{r^6}\right)\right]\right]^{N-1} \\
&\xrightarrow{N\to\infty} \exp\left(\int \mathrm{d}\Delta_k\, \bar{n}(\Delta_k)\frac{N}{V}\frac{1}{4}\sum_{O_k}\int \sin(\theta)\,\mathrm{d}\theta\,\mathrm{d}\varphi \int r^2\,\mathrm{d}r \times\right. \\
&\qquad\qquad \left.\times\left[1-\exp\left(-\frac{4\gamma t[g_{ki}^2+h_{ki}^2]}{(\Delta_k-\Delta_i)^2+4\gamma^2}\frac{J_0^2}{r^6}\right)\right]\right) \\
&= \exp\left(\frac{N}{V}\frac{4\pi^{\frac{3}{2}}}{3}\int \mathrm{d}\Delta_k\, \bar{n}(\Delta_k)\frac{1}{4}\sum_{O_k}\frac{1}{4\pi}\int \sin(\theta)\,\mathrm{d}\theta\,\mathrm{d}\varphi\sqrt{\frac{4\gamma t J_0^2[g_{ki}^2+h_{ki}^2]}{(\Delta_k-\Delta_i)^2+4\gamma^2}}\right) \\
&=: \exp\Big(-\sqrt{t/T_{\rm r}(\Delta_i)}\Big),
\end{aligned} \tag{S23}$$

where we defined

$$T_{\rm r}^{-1}(\Delta) := \left(\frac{N}{V}\right)^2 \frac{J_0^2}{\gamma}\frac{16\pi^3}{9}\nu^2(\Delta)\xi^2(O), \tag{S24}$$

$$\nu(\Delta) = \int \mathrm{d}\Delta'\,\bar{n}(\Delta')\sqrt{\frac{4\gamma^2}{(\Delta'-\Delta)^2+4\gamma^2}}, \tag{S25}$$

$$\xi(O) = \frac{1}{4}\sum_{O'}\zeta_{OO'}, \quad \zeta_{OO'} = \frac{1}{4\pi}\int \sin(\theta)\,\mathrm{d}\theta\,\mathrm{d}\varphi\sqrt{g_{OO'}^2+h_{OO'}^2}. \tag{S26}$$

Note that we write $g_{OO'}$ and $h_{OO'}$ with orientation indices, since they only depend on the orientation and the angle, but not on the spin-spin distance. The angular average is parametrised in spherical coordinates, i.e., $\hat{\mathbf{u}}$ is replaced by the unit vector in spherical coordinates in Eq. (S14) before the average. Different orientations O sample the corresponding coordinate systems from Eqs. (S8) and (S9). For the angular average over different NV^- orientations, we find

$$(\zeta_{OO'}) = \begin{pmatrix} 0.38 & 0.65 & 0.65 & 0.83 \\ 0.65 & 0.38 & 0.83 & 0.65 \\ 0.65 & 0.83 & 0.38 & 0.65 \\ 0.83 & 0.65 & 0.65 & 0.38 \end{pmatrix}. \tag{S27}$$

Note that it was already observed in [1] that, on average, the relaxation of spins within one subensemble of a specific orientation is lower than the inter-subensemble relaxation. For our specific setup with 4 subensembles on resonance, we find that there is a further distinction between different inter-subensemble relaxation rates. Namely, for each orientation, there exist two different orientations with relative relaxation weight 0.65 and one with relative relaxation weight 0.83 [cf. Eq. (S27)]. This can be understood when considering that for a chosen quantisation axis in Fig. S1b, two other (directed) quantisation axes enclose an angle of 70.5° with that axis while the third axis encloses an angle of 109.5°. That way, we find $\xi^2 = 0.397$ for the angular average. The frequency average is determined from the experimental spin distribution; on resonance, we arrive at $\nu^2(\Delta = 0) = 0.0480$. Using the parameters of Fig. 3 in the main text, we thus find $T_\mathrm{r}(\Delta = 0) = 1.14\,\mu\mathrm{s}$. While the on-resonance relaxation of the inversion also follows a stretched exponential, the experiment yields a considerably longer relaxation timescale of about $T_\mathrm{r} = 11.6\,\mu\mathrm{s}$ (see Fig. 2 in the main text). Note that, although the timescales are quite different, we find that some features of the relaxation persist qualitatively in the full simulation. First, the on-resonance population relaxes with a stretched exponential behaviour. Second, we find the on-resonance relaxation rate to be the same for different initial inversions p_0 before the first superradiant decay, and thus different shapes of the spectral hole. This is evident in the approximate solution, where the different frequencies relax completely independently of each other and is consistent with the observations in Extended Data Fig. 2.

[1] Choi, J. *et al.* Depolarization dynamics in a strongly interacting solid-state spin ensemble. *Physical Review Letters* **118**, 093601 (2017).
